# Quantitative performance assessment of Ultivue multiplex panels in formalin-fixed, paraffin-embedded human and murine tumor specimens

**DOI:** 10.1038/s41598-024-58372-5

**Published:** 2024-04-11

**Authors:** Sripad Ram, Sepideh Mojtahedzadeh, Joan-Kristel Aguilar, Timothy Coskran, Eric L. Powell, Shawn P. O’Neil

**Affiliations:** 1 Drug Safety Research and Development, Pfizer Inc., Groton, CT USA; 2 Oncology Research and Development, Pfizer Inc., San Diego, CA USA

**Keywords:** Tumour biomarkers, Predictive markers, Prognostic markers

## Abstract

We present a rigorous validation strategy to evaluate the performance of Ultivue multiplex immunofluorescence panels. We have quantified the accuracy and precision of four different multiplex panels (three human and one mouse) in tumor specimens with varying levels of T cell density. Our results show that Ultivue panels are typically accurate wherein the relative difference in cell proportion between a multiplex image and a 1-plex image is less than 20% for a given biomarker. Ultivue panels exhibited relatively high intra-run precision (CV ≤ 25%) and relatively low inter-run precision (CV >> 25%) which can be remedied by using local intensity thresholding to gate biomarker positivity. We also evaluated the reproducibility of cell–cell distance estimates measured from multiplex images which show high intra- and inter-run precision. We introduce a new metric, multiplex labeling efficiency, which can be used to benchmark the overall fidelity of the multiplex data across multiple batch runs. Taken together our results provide a comprehensive characterization of Ultivue panels and offer practical guidelines for analyzing multiplex images.

## Introduction

Tumor tissue is a complex microenvironment that involves the interactions of multiple phenotypes of immune cells whose abundance and spatial organization can impact prognosis and treatment outcome^1,2^. Immune cells are highly dynamic, and the unambiguous identification of their phenotypes typically requires the presence of multiple biomarkers. Thus, the need for tissue multiplexing techniques naturally arises in this context as it enables the simultaneous detection and localization of several biomarkers in tissue sections. The advent of immunotherapy has led to the development of numerous multiplexing technologies, which are increasingly being used to infer spatial patterns of immune cell infiltration and to gain insights into mechanisms of action or resistance to the drugs in development^3–6^.

Multiplexing techniques can be classified in several ways. One approach is to use readout method as a means of categorization, i.e., fluorescence, chromogenic, or heavy metal-tagged techniques. Alternately, these techniques can be categorized based on workflow. For instance, some techniques make use of a cyclic workflow of immunolabeling, slide scanning and stripping, with the process repeated numerous times until the desired number of biomarkers are labeled and imaged. Examples of this workflow include MELC^7^, MxIF^8^, CODEX^9^, MICSSS^10^, CyCIF^11^, SeqStain^12^ and MIBI^13^. Other approaches use a modular workflow, wherein sample labeling and slide scanning are distinct steps that don’t involve a repetitive workflow. Examples of this workflow include Ultivue^14^, CellIdx^15^, SignalStar™^16^ and Opal-TSA panels^17^. Common to all these techniques is the image analysis workflow, which involves nuclear segmentation, cell phenotyping and additional downstream analyses such as measurement of cell–cell distances, neighborhood analysis, etc.

Given the breadth of multiplexing techniques that are commercially available, our choice of Ultivue InSituPlex (ISP) technology was based on several considerations. First, we sought a platform technology with broad scope of use with targets. ISP is a modular platform technology in which cocktails of antibodies, each conjugated with a unique DNA barcode, are applied to tissue sections, followed by reporter fluorophores conjugated with complementary DNA barcodes to create a 4-plex panel. More recently, ISP technology has been extended to 8- and 12-plex panels by repetitive sequences of 4-plex labeling, scanning and reporter fluorophore removal (using dehybridization washes to disassociate the DNA barcodes) on the same tissue section. Next, compatibility with a traditional pathology laboratory workflow was also essential where immunolabeling, slide scanning and image analysis are non-overlapping, distinct tasks. A relatively low cost of adoption ensured that investment in new capital equipment was not required, as existing laboratory equipment (e.g., autostainers, slide scanners, etc.) and software (for image analysis) could be used. ISP panels are offered as ready to use kits for the Leica Bond Rx automated immunolabeling instrument which makes it easy to deploy these assays even in a regulated (e.g. CLIA) environment. The barcode-conjugated fluorophores used have distinct spectral separation and thus can be scanned using any fluorescence slide scanner that is capable of imaging in the 400–800 nm wavelength range. Finally, operating cost and throughput were compatible with use in large studies. The latter was especially important in a drug discovery and development environment where most studies have short turnaround times for data delivery.

The concordance and reproducibility of multiplex imaging data is a fundamental concern when using multiplexing techniques. In a multiplex assay, a single tissue section is incubated with multiple antibodies directed against multiple biomarkers, some or all of which may label one or very few cell types. Thus, a question arises as to whether the labeling efficiency for a given biomarker is impacted (due to steric hindrance of antibodies, for example) when the specimen is simultaneously incubated with multiple biomarkers. Consequently, it is important to ascertain whether the expression of a given biomarker in the multiplex image is concordant to that of a single-plex image labeled for only that specific biomarker. In many applications, tumor specimens are assayed in multiple batches; thus, reproducibility of the multiplexing technique is of great significance, as it informs on the reliability of the endpoints calculated from the multiplex images generated throughout an experiment^18^.

Prior studies have investigated the precision of Opal-TSA^18–20^, MIBI-TOF^21^ and CyCIF^22^ technologies. These reports used serial sections of tissue microarrays as the test substrate and evaluated precision by quantifying correlations in cell count, cell density or % positive cells between runs. Each of these studies reported acceptable correlations among assays for the biomarkers evaluated; thus demonstrating high reproducibility in detecting various cell phenotypes. There have also been numerous reports that provide best practices and guidelines for the design and optimization of multiplexing panels for use in tumor biopsy sections^23–26^. Specifically, these reports recommend quantitative assessment of reproducibility of the multiplex panels within and across multiple batch runs.

Here we provide a rigorous assessment of the concordance (i.e., accuracy) and reproducibility (i.e., precision) of Ultivue ISP technology. We have validated a custom 4-plex panel for murine tissue and three commercially available “off-the-shelf" human 4-plex panels for human tissue. The custom mouse panel was designed to detect T cell biomarkers CD3ε, CD4, CD8α and FoxP3 and was evaluated on murine tumor specimens with varying levels of T cell infiltration. The three off-the-shelf panels for human tissue were Tact (CD3, Ki67, Granzyme B and PanCK), PD-L1 (CD8, CD68, PD-L1 and PanCK) and APC (CD11c, CD20, CD68+CD163 cocktail and MHCII) panels, which were evaluated on human breast tumor specimens with varying levels of T cell density.

Results from the concordance experiments showed that for all panels evaluated, the relative difference in the proportion of positive cells between the 4-plex image and the corresponding 1-plex image for a given biomarker is typically less than 20%. For the mouse panel, results from the precision study revealed relatively low intra-run coefficient of variation (CV) in the proportion of positive cells (i.e., variability is typically < 25%); whereas inter-run CV was typically well above 25%. The latter was due to batch-to-batch variability where there was considerable variation in the intensity of labeling of biomarkers across different runs. Reanalysis of the precision data using local thresholding for biomarker positivity within a run resulted in improved inter-run CV without affecting the intra-run CV. Results of the precision study for the human panels also revealed relatively low inter-run CV for most of the biomarkers tested when a local thresholding approach was utilized for detecting biomarker positivity. Finally, we evaluated the reproducibility of spatial distance estimates for the mouse panel, which showed relatively high intra- and inter-run precision in two different tumor models.

We introduce a new metric, multiplex labeling efficiency, to empirically benchmark the fidelity of a multiplex dataset. The motivation behind introducing such a metric is to enable comprehensive assessment of data quality when multiplex images are generated from multiple assay runs. Such situations arise, for example, when analyzing clinical trial specimens where tissue sections are made available at different times, or when comparing data generated using the same multiplex panel from repeat experiments. Multiplex labeling efficiency for the mouse panel revealed relatively high variability across multiple batch-runs when the data was analyzed using global thresholding for biomarker positivity. Interestingly, this variability is diminished when multiplex labeling efficiency is computed from data that is analyzed using local thresholding for biomarker positivity.

Our results provide a comprehensive assessment of the performance of Ultivue ISP technology and offer a template for validating multiplex panels. In addition, our analyses also provide practical guidelines for quantifying multiplex data fidelity and for analyzing multiplex data.

## Materials and methods

### Human and murine tumor specimens

No humans were directly involved in this study. All human tissue biospecimens used in the study were anonymized specimens that were acquired by Pfizer from Indivumed Services (Frederick, MD). These specimens were used in compliance with Pfizer’s policy on the Use of Human Biological Specimens^27^. Specifically, these biospecimens were collected with written patient consent, processed, and distributed in full ethical and regulatory compliance with the sites from which they were collected. This includes independent ethical review, Institutional Review Board approval (where appropriate), and independent regulatory review. All animal procedures were compliant with the Guide for the Care and Use of Laboratory Animals^28^ and were approved by the Pfizer Global Research and Development Institutional Animal Care and Use Committee. All animal experiments were reported in accordance with the ARRIVE guidelines^29^. For evaluating human multiplex panels, we selected 4 different cases of triple negative breast cancer resections with high, medium or low levels of T cell density in the tumor and stromal regions.

For the mouse panel, we selected CT26, 4T1 and B16F10 tumor models which are known to have relatively high, medium and low levels of T cell density, respectively. CT26, 4T1 and B16F10 cell lines were purchased from ATCC (Manassas, VA). Female BALB/c mice and female C57BL6 mice were purchased from Jackson Laboratory. CT26 (2 × 10^5^ cells) or 4T1 (1 × 10^5^ cells) cells were implanted subcutaneously and in the mammary fat pad, respectively, in BALB/c mice. B16F10 cells (5 × 10^5^ cells) were implanted subcutaneously in C57BL6 mice. Once tumors reached the desired size (CT26 and 4T1 tumors: 300–400 mm^3^; B16F10 tumor: 200–300 mm^3^) mice were euthanized using 4% isoflurane and the tumors were collected. The murine tumor samples were fixed in 10% neutral buffered formalin for 48 h at room temperature then trimmed and embedded in paraffin blocks.

### Antibody validation

The antibodies against murine biomarkers were validated using a panel of tissue specimens with varying levels of biomarker expression. Briefly, each antibody was initially tested using the recommended vendor protocol and the immunolabeling conditions (epitope retrieval buffer, antibody dilution, incubation time and temperature) were systematically varied. After each round of optimization, the slides were reviewed by a pathologist who evaluated for correct regional distribution (e.g., CD3ε immunoreactivity predominantly in T-cell follicles in spleen) and subcellular localization pattern of immunoreactivity (e.g., CD3ε in the cell membrane). Slides with weak on-target signal, off-target or non-specific labeling were flagged and the immunolabeling conditions were further optimized to mitigate these effects.

### Immunohistochemistry

Chromogenic IHC assays were performed on 5-micron thick FFPE sections with one of the following antibodies against mouse antigens using the Leica Bond III automated IHC instrument (Leica Biosystems, Buffalo Grove, IL): rabbit anti-CD3ε (clone D4V8L, 1:75 dilution or 0.57 µg/ml; Cat# 99940, Cell Signaling Technology, Danvers, Cambridge, MA), rabbit anti-CD4 (clone D7D2Z, 1:1500 or 0.103 µg/ml; Cat# 25229, Cell Signaling Technology), rat anti-FoxP3 (clone FJK-16s, 1:200 or 2.5 µg/ml; Cat# 14-5773-82, Thermo Fisher, Waltham, MA) and rabbit anti-CD8α (clone D4W2Z, 1:400 or 1.59 µg/ml, Cat# 98941, Cell Signaling Technology). Tissue sections for CD8α, CD4, and FoxP3 were loaded onto the Leica Bond instrument for deparaffinization followed by epitope retrieval using Leica Epitope Retrieval Solution 2 (Leica Biosystems) for 20 min. Tissue sections for CD3ε staining were first placed in Borg epitope retrieval solution (Biocare Medical, Pacheco, CA) and incubated in Biocare’s Decloaking Chamber at 125° for 5 min. Slides were removed from the chamber, rinsed in Leica Bond Wash and loaded onto the Leica Bond instrument. CD3ε and CD4 antibodies were incubated at room temperature for 60 min, while CD8α and FoxP3 were incubated at 30 min. CD3ε, CD4, and CD8α were detected using Leica Bond Refine DAB polymer (Leica Biosystems), while FoxP3 used an additional rabbit anti-rat linker antibody (Vector Laboratories, Newark, CA) prior to the Refine DAB polymer. All slides were counterstained with Hematoxylin included in the Refine DAB polymer kit.

For multiplex panels, 5-micron thick unstained, cut section were shipped to Ultivue, where the slides were loaded onto a Leica Bond Rx automated IHC instrument (Leica Biosystems) for 4-plex immunolabeling. The slides were counterstained with DAPI, which was included in the 4-plex kit. To minimize pre-analytic variability, for a given panel the same Leica Bond Rx instrument and reagents from the same lot were used for immunolabeling.

### Fluorescence slide scanning

All multiplex slides were scanned using a Zeiss Axioscan.Z1 fluorescence slide scanner (Carl Zeiss, Jena, Germany) equipped with a LED light source, 20 × 0.8 NA planApochromat objective lens and an ORCA FLASH scientific complementary metal oxide semiconductor camera (Hamamatsu Corp, Hamamatsu, Japan). The images were stored in CZI file format.

### Whole slide image analysis

For the mouse panel, whole-slide image analysis of concordance and precision images were independently performed by two image analysis scientists (S.R. and S.M.) using QuPath version 0.3.2^30^ and the Highplex FL module in HALO version 3.3 (Indica Labs, Albuquerque, NM). Both software packages yielded very similar proportion of single- and multi-marker cells (data not shown). Briefly, the image analysis workflow consisted of the following steps: (1) manual outlining of the tissue of interest and exclusion of necrotic regions, tissue folds, and host-connective tissue, (2) nuclear segmentation using the default segmentation algorithm, (3) estimation of cell boundary by dilating the nuclear boundary by a preset distance (5 microns), (4) determine cell positivity for each biomarker based on a threshold value for the average intensity of that biomarker in the cell, (5) phenotype cells based on positivity of multiple biomarkers. No correction for membrane spillover was performed.

For consistency, image analysis results from QuPath are shown throughout the manuscript. For the human panels, whole-slide image analysis of the concordance and precision images was performed using QuPath version 0.2.3. For both human and mouse concordance images, the mean intensity/cell of every biomarker for all cells was exported to MATLAB programming language (MathWorks, Natick, NA), which was used to calculate the average intensity of each biomarker for the 1-plex and 4-plex images. Cell–cell distance estimation in mouse precision images was carried out using the Spatial Analysis module in HALO version 3.4. Specifically, we considered cytotoxic T cells (CD3ε+CD8α+ cells) and regulatory T cells (CD3ε+CD4+FoxP3+ cells) and calculated the nearest neighbor distances for these two cell types.

In the mouse panel, for global thresholding a single threshold value was arbitrarily set in the image analysis workflow to determine positive cells for a given biomarker for all the images from different runs. This threshold was determined by examining multiple fields of view across images from different runs. The thresholds were selected in a stringent manner to minimize spurious detection of positive cells due to autofluorescence and lateral spillover. For local thresholding, a separate threshold value was calculated using the method of Otsu^31^ for each run to determine positive cells for a given biomarker. Here, the image analysis workflow (QuPath) consisted of nuclear segmentation and cell boundary detection. Mean biomarker intensities in all channels for all detected cells were then exported to MATLAB where Otsu thresholding (multithresh method) was performed for each biomarker using data from within a run.

Multiplex labeling efficiency (MLE) for the mouse markers are defined as follows:

$$ {\text{MLE}}\,\,{\text{for}}\,\,{\text{CD}}3\varepsilon \, = \,100 \times \,\frac{{\# {\mkern 1mu} \,T_{{{\text{cyt}}}} \,{\text{cells}}\, + \,\# \,{\mkern 1mu} {\text{of}}\,{\text{ }}T_{{{\text{helper}}}} \,{\text{cells}}\, + \,\# {\mkern 1mu} \,{\text{of }}\,T_{{{\text{reg}}}} \,{\text{cells}}}}{{{\text{Tot}}\,\# \,{\text{of }}\,{\text{CD}}3\varepsilon  + {\text{cells}}}},   $$

$$   {\text{MLE}}\,\,{\text{for}}\,\,{\text{CD}}4\, = \,100\, \times \,\frac{{\# \,{\text{of}}\,\,T_{{{\text{helper}}}}\, {\text{cells}}\, + \,\# {\mkern 1mu} \,{\text{of}}\,{\mkern 1mu} T_{{{\text{reg}}}} {\mkern 1mu} \,{\text{cells}}}}{{{\mkern 1mu} {\text{Tot}}\,\# {\mkern 1mu} \,\,{\text{of}}\,{\text{CD}}4\, + \,{\text{cells}}}},  $$

$$ {\text{MLE}}\,\,{\text{for}}\,\,{\text{CD8}}{\mkern 1mu} \alpha \, = \,100\, \times \,\frac{{\# \,\,T_{{{\text{cyt}}}} {\mkern 1mu} \,\,{\text{cells }}}}{{{\text{Tot}}\,\,\# \,\,{\text{of}}\,\,{\text{CD}}8{\mkern 1mu}\alpha  + \,{\text{cells}}}}, $$

$$ {\text{MLE }}\,\,{\text{for }}\,{\text{Fox}}\,{\text{P3}} = 100\, \times \,\frac{{\# {\mkern 1mu} \,{\text{of}}\,\,{\mkern 1mu} T_{{{\text{reg}}}} {\mkern 1mu} {\text{ }}\,{\text{cells}}}}{{{\text{Tot}}\,\# {\mkern 1mu} \,{\text{of}}{\mkern 1mu} \,{\text{Fox}}\,{\mkern 1mu} {\text{P3}}\, + \,{\text{cells}}}}, $$

where T_cyt_ denotes CD3ε+CD8α+ cells, T_helper_ denotes CD3ε+CD4+ cells and T_reg_ denotes CD3ε+CD4+FoxP3+ cells.

## Results

### Design of a custom mouse 4-plex panel

**Figure 1:**
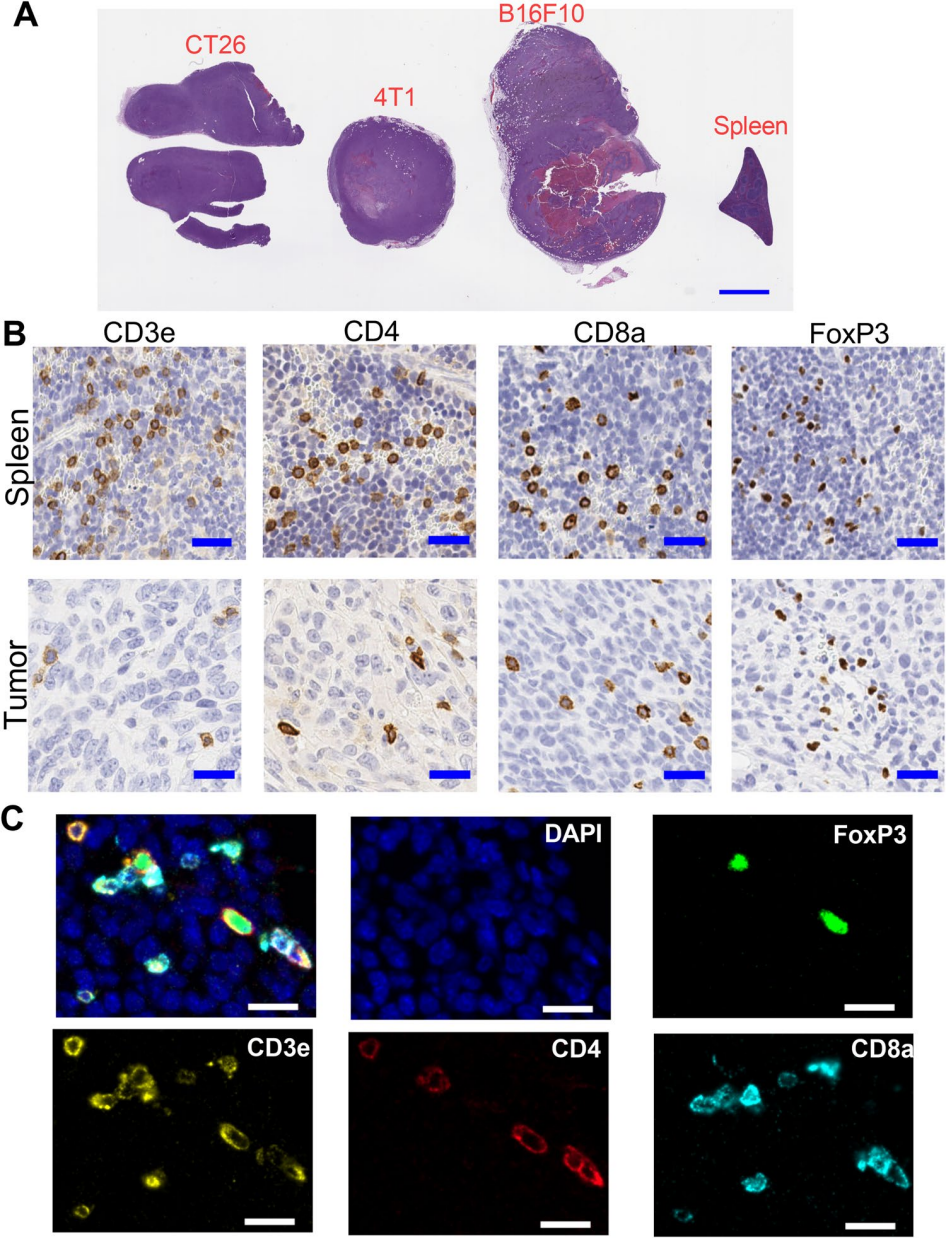
Murine multi-tissue block & IHC images. (**A**) Shows an image of an H&E stained section from a multi-tissue block, containing CT26, 4T1 and B16F10 tumors and a transverse section of normal mouse spleen. Scale bar equals 2 mm. (**B**) Shows representative IHC images of CD3ε, CD4, CD8α and FoxP3 in normal mouse spleen (top row) and in CT26 tumor (bottom row). Scale bar equals 25 microns. (**C**) Shows merged (upper left) and individual immunofluorescence channels of CT26 tumor that was immunolabeled with the murine multiplex panel. Scale bar equals 25 microns.

We designed a custom T cell panel to detect CD3ε, CD4, CD8α and FoxP3 in murine tissue to characterize T cell infiltrates in murine syngeneic tumors and autoimmune disease models. As test substrate we created a multi-tissue block consisting of a transverse tissue section of mouse spleen and tumor tissue from three different murine syngeneic tumor models (CT26, 4T1 and B16F10) with varying levels of T cell density (Fig. 1A). The specific tumors selected for inclusion in the multi-tissue block were specimens with limited amounts of necrosis and good cell viability. Next, we validated antibody clones through immunohistochemistry (IHC) that recognized the desired murine T cell antigens in formalin-fixed, paraffin embedded (FFPE) sections of murine spleen. We chose antibody clones that showed appropriate regional (e.g., splenic white pulp) and subcellular localization (e.g., cell membrane for CD3ε, CD4 and CD8α and nucleus for FoxP3) in spleen and tumor tissue (Fig. 1B). The selected antibody clones were conjugated to unique DNA barcodes, which were then used to construct the 4-plex T cell panel.

In Ultivue’s InsituPlex immunofluorescence (IF) assay, deparaffinized FFPE tissue sections are first subjected to an epitope retrieval step and then incubated with a cocktail containing DNA barcoded antibodies. This is followed by a signal amplification step that involves the elongation of the DNA barcodes to create additional “touchdown” sequences. Finally, a mixture of detection probes is added which consists of distinct fluorescent labels conjugated with DNA barcodes that are complementary to the barcode sequences on the antibodies. The unique specificity of the barcode sequences paired with the presence of multiple hybridization sites for the detection probes on each antibody barcode generates a strong fluorescence signal from the target cell expressing the biomarker of interest. Figure 1C shows a fluorescence image of a CT26 tumor that was labeled with the custom 4-plex panel, demonstrating robust membrane localization for CD3ε, CD4 and CD8α biomarkers and nuclear localization for FoxP3, consistent with the subcellular localization pattern that was observed in brightfield IHC images (Fig. 1B).

### Concordance assay for mouse Ultivue panel

**Figure 2:**
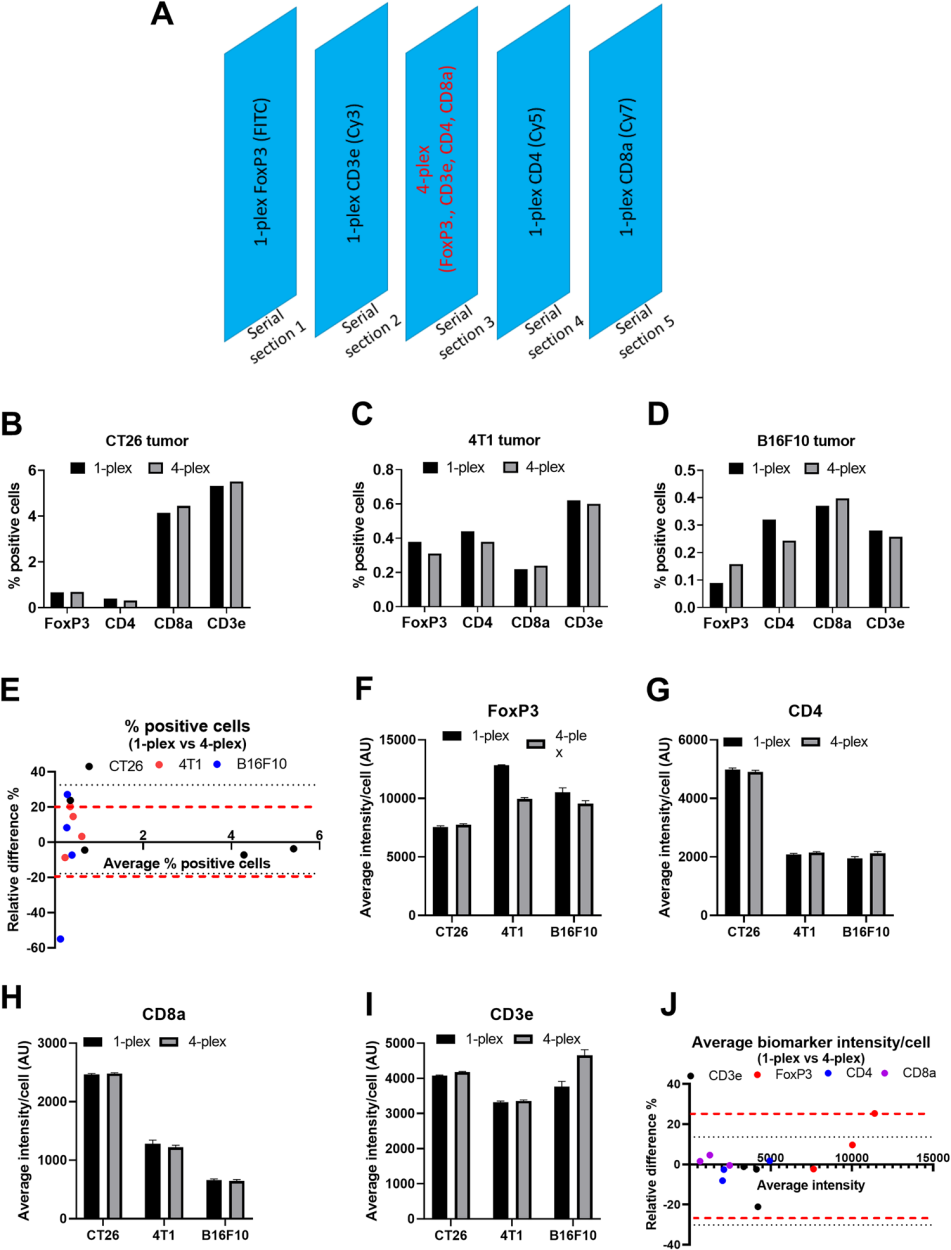
Murine 4-plex panel: concordance assay. (**A**) Shows the design of the concordance assay for the murine 4-plex panel. Five serial sections are cut from the multi-tissue block. The third section is immunolabeled with the 4-plex assay while the remaining sections are 1-plex assays immunolabeled for CD3ε, CD4, FoxP3 and CD8α. (**B**), (**C**) and (**D**) Show % positive cells quantified through whole-slide image analysis from the 1-plex and 4-plex images of CT26, 4T1 and B16F10 tumors, respectively. (**E**) Shows a Bland–Altman plot of the relative difference in % positive cells between the 1-plex image and the 4-plex image for all the murine tumor models. Here each data point pertains to one biomarker and the color pertains to the tumor model type. (**F**), (**G**), (**H**) and (**I**) Show the average intensity/cell of FoxP3, CD4, CD8α and CD3ε biomarkers, respectively, in the 1-plex and 4-plex images for different murine tumor models. (**J**) Shows a Bland–Altman plot of the relative difference in the average intensity/cell for the different biomarkers across all three murine tumor models. Here each data point pertains to one biomarker and the color pertains to the tumor model type.

In many multiplex panels, including our T cell panel, two or more biomarkers are typically expressed on the same cell type and sometimes even in the same subcellular location. Thus, a valid concern is whether the binding specificity of a particular antibody is impacted by the close proximity of other antibodies (due to steric hindrance, for example). To address this question, we designed a concordance assay using five adjacent serial sections from our multi-tissue block (Fig. 2A). The third serial section was immunolabeled through the 4-plex assay and the remaining serial sections were immunolabeled with the corresponding 1-plex IF assays for the four different biomarkers. The 1-plex and 4-plex images were subjected to whole-slide digital image analysis to quantify the % positive cells for each biomarker. Figure 2B–D show the results of the analyses for three different tumor models, in which we found tight agreement in the % positive cells between 1-plex and 4-plex images for each of the four biomarkers tested. Figure 2E shows a Bland–Altman plot of the relative difference in % positive cells between 1-plex and 4-plex images which is typically within ± 20% for all four biomarkers across the three different tumor models. A high relative difference of ~ 60% was observed for FoxP3 in the B16F10 tumor model. This can be attributed to the very low abundance of this biomarker in a cold tumor model, resulting in high variability in % positive cells between serial sections used for the 1-plex and 4-plex images.

As the detection of positive cells is primarily based on the intensity of the biomarker, we also compared the mean intensity/cell of the positive cells between the 1-plex and the 4-plex image for each biomarker across the three different tumor models. We and found that the average intensity/cell was similar between the 1-plex and 4-plex images for each of the four biomarkers (Fig. 2F–I; also see supplementary Table 1 for summary table). Interestingly, for CD4 and CD8α the average intensity/cell is consistently lower in 4T1 and B16F10 tumor models relative to the CT26 tumor model. We speculate that this could partly be due to the relative location of the 4T1 and B16F10 tumor sections on the slide with respect to the CT26 tumor section (Fig. 1A). Bland Altman analysis of the biomarker intensity shows that the relative difference between 1-plex and 4-plex images was typically less than 20% for all biomarkers and across all three tumor models (Fig. 2J). These results suggest that there is robust concordance between 1-plex and 4-plex images from the three tumor models.

### Precision assay for mouse Ultivue panel

**Figure 3:**
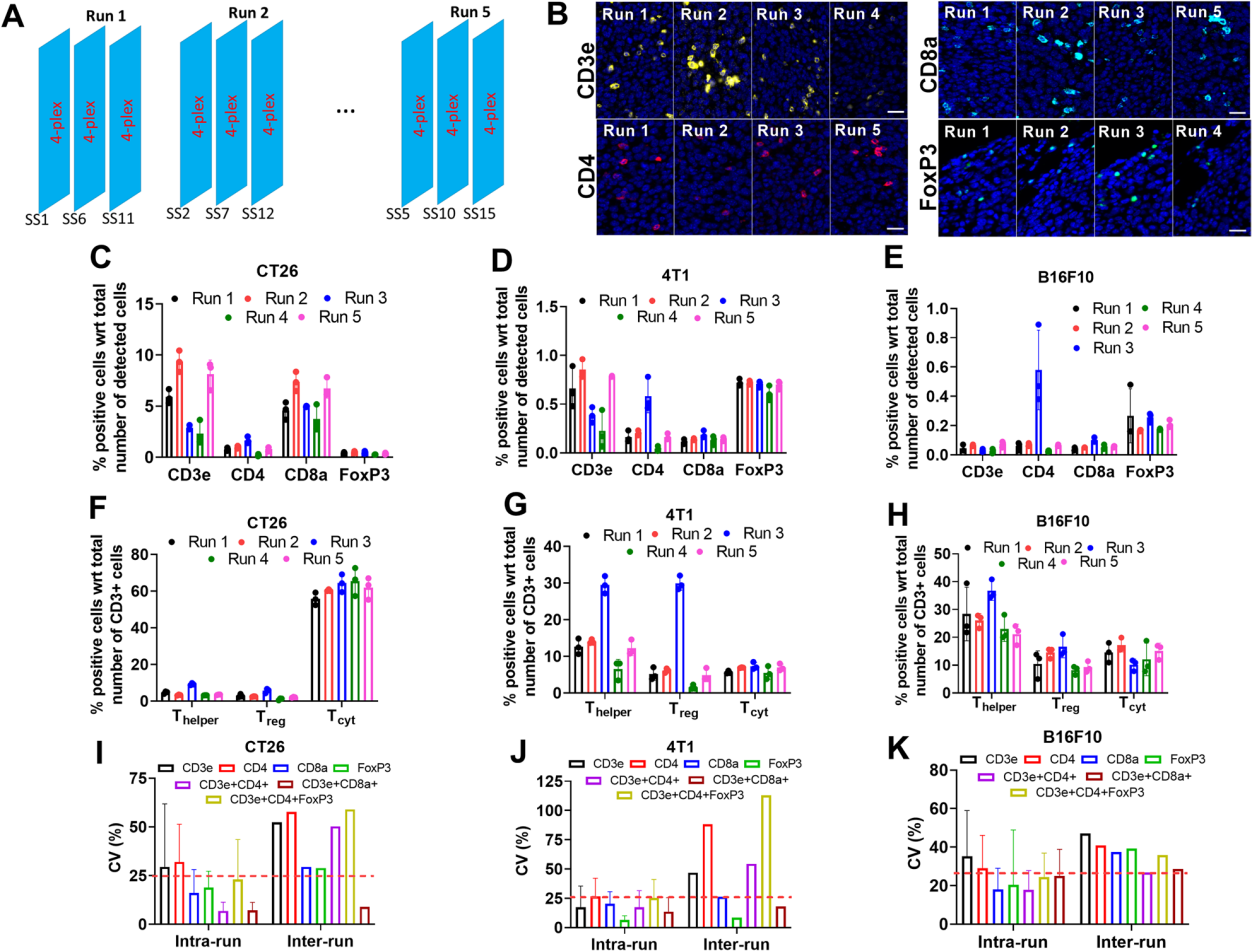
Murine 4-plex panel: reproducibility assay. (**A**) Shows the design of the reproducibility assay. Fifteen serial sections were cut from the multi-tissue block and immunolabeled with the murine 4-plex panel in 5 separate runs with 3 replicates per run. (**B**) Shows representative fluorescence images of the biomarkers across the different runs. Scale bar equals 50 microns. (**C**), (**D**) and (**E**) Show % positive cells of single-marker cell phenotypes for CT26, 4T1 and B16F10 tumor models, respectively, while (**F**), (**G**) and (**H**) show the same for multiple-marker cell phenotypes (T_helper_: CD3ε+CD4+ cells; T_reg_: CD3ε+CD4+FoxP3+ cells; T_cyt_ denotes CD3ε+CD8α+ cells). (**I**), (**J**) and (**K**) Show the intra-run and inter-run CV for single-marker and multi-marker cell phenotypes for CT26, 4T1 and B16F10 tumor models, respectively.

Figure 3A shows the design of our precision assay, in which the 4-plex panel was run in triplicate on five separate days. For this assay, adjacent serial sections of the multi-tissue tumor block were shuffled such that serial sections 1, 6 and 11 were used for run 1, serial sections 2, 7 and 12 were used for run 2, and so forth. In this way, we avoided any systematic drifts in quantifying biomarker expression due to serial sectioning effect.

To assess reproducibility of the 4-plex assay, we detected and counted the number of CD3ε, CD4, CD8α and FoxP3 positive cells, i.e., the single-marker cell phenotypes. In addition, we also detected three multi-marker cell phenotypes, i.e., CD3ε+CD8α+ cytotoxic T cells (T_cyt_), CD3ε+CD4+ helper T cells (T_helper_) and CD3ε+CD4+FoxP3+ regulatory T cells (T_reg_). We used a cutoff value of 25% for CV which is consistent with the range of cut off values used in the literature for assessing reproducibility of IHC assays^32–35^. Figure 3B shows representative images for the four different biomarkers from different runs. Note that there is considerable variation in the signal intensity for any given biomarker across different runs. Figure 3C shows the % positive cells for the single-marker cell phenotypes in the CT26 tumor model across five different runs. The % positive cells for each biomarker was relatively consistent within individual runs, but significant variability was observed across separate runs. For example, for CD3ε, the mean % positive cells varies from 9.4% (run 2) to 2.3% (run 4) across the different runs (see supplementary Table 2 for summary table of plots). This variability can be attributed to differences in the fluorescence intensity observed for each of the biomarkers across different runs, as shown in Fig. 3B. Similarly, in the 4T1 (Fig. 3D) and B16F10 (Fig. 3E) tumor models, good agreement in % positive cells is typically observed within a given run, but considerable variability exists between runs. Note that in 4T1 and B16F10 tumor models, the relative abundance of FoxP3+ cells is higher than that of CD4+ and CD3ε+ cells in most runs. This can be partly attributed to the fact that in some tumor models FoxP3 is expressed in other lymphoid and myeloid cells, and in some non-hematopoietic cells such as cancer cells^36–39^.

Figure 3F–H show the % positive cells for multi-marker cell phenotypes across different runs for all three tumor models. Analogous trends were observed here as well, in which % positive cells were consistent within a run, but considerable variation existed across different runs. Figure 3I–K show the intra- and inter-run CV for the single and multi-marker cell phenotypes for all three tumor models. Consistent with the observations described above, the intra-run CVs (i.e., CV of % positive cells within an assay) were typically less than 25% for all cell phenotypes; however, the inter-run CV (i.e., CV of % positive cells across all the assays) is typically high (> 25%) for all cell phenotypes. These observations suggest that run-to-run variability is the primary source of variability in % positive cells for this multiplex panel.

### Local thresholding improves run-to-run variability

**Figure 4:**
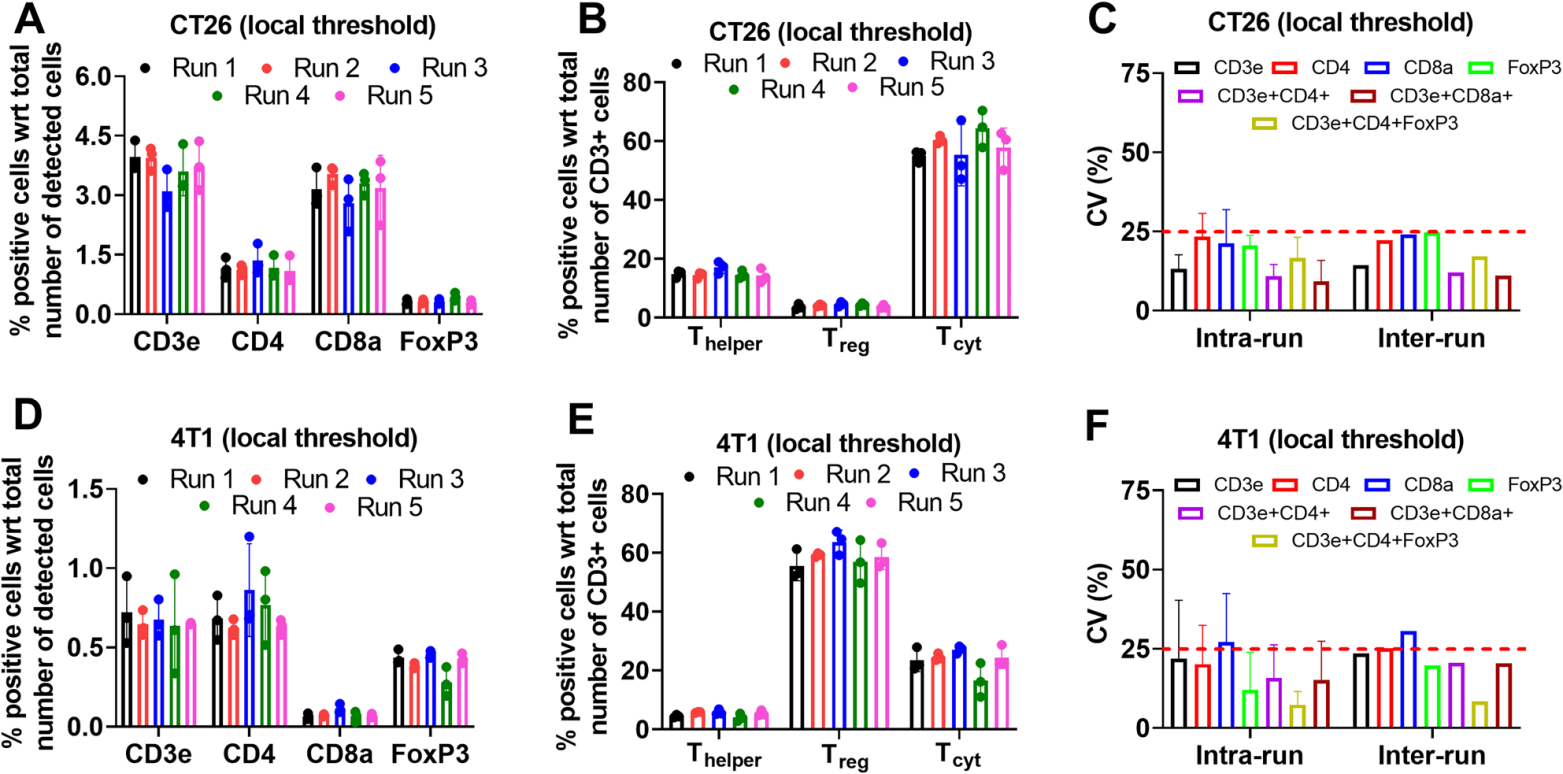
Local thresholding decreases inter-run variability without affecting intra-run variability. (**A**) and (**B**) Show the % positive cells for single-marker and multi-marker cell phenotypes, respectively, for the CT26 murine tumor model across different runs. (**C**) Shows the intra-run and inter-run CVs of single- and multi-marker cell phenotypes for the CT26 tumor model. (**D**), (**E**) and (**F**) Show the % positive cells for single-marker (**D**) and multi-marker (**E**) cell phenotypes, and intra-run and inter-run CVs of single- and multi-marker cell phenotypes (**F**) for the 4T1 tumor model.

For the precision analysis described above, we adopted a global thresholding strategy where positivity of a particular biomarker was based on an intensity cutoff value which was fixed for all of the images. As the multiplex images showed considerable batch-to-batch variation, we hypothesized that a local thresholding strategy might be beneficial. Consequently, we repeated the precision study analysis using local thresholding; for a given biomarker we used a different intensity cutoff for each batch run, which was specifically determined by Otsu thresholding (see methods for details). Figure 4A,B show the % positive cells for single marker and multi-marker cell phenotypes based on local thresholding for the CT26 tumor model (also see Supplementary Table 3 for summary table). Consistent with Fig. 3C,F, we see that the % positive cell estimates within a run are very close to one another. Interestingly, and in contrast to Fig. 3C,F, the % positive cells estimates are also tight across different runs. Consistent with these observations, calculations of the intra-run and inter-run CVs were typically below 25% for all cell phenotypes (Fig. 4C). Similar results were also seen for the 4T1 tumor model (Fig. 4D,E) where intra- and inter-run CVs were below 25% for all cell phenotypes (Fig. 4F). These results suggest that local thresholding may be a more beneficial approach to get robust cell proportion estimates despite batch-to-batch variability in the multiplex images. We also applied local thresholding to B16F10 tumor images, but this did not improve run-to-run variability (data not shown). We hypothesize that this lack of improvement is due to the very low T cell counts in B16F10 tumors, which inherently introduces greater variability across serial sections which was not resolved with local intensity thresholding.

### Multiplex labeling efficiency—a metric to assess fidelity of multiplex data

The analysis described above shows how the calculation of inter- and intra-run CV is useful in assessing assay variability. However, in many practical situations it is not feasible to calculate inter- and intra-run CVs, since this will require the inclusion of a positive control tissue in triplicate which can be expensive. Thus, we propose a new metric, multiplex labeling efficiency (MLE), which can be interpreted as an integrated measure of performance of the IF assay and the image analysis workflow. MLE is defined for each biomarker and is calculated as the fraction (expressed as a percentage) of cells that are positive for two or more valid biomarker combinations including the biomarker of interest. For example, for the mouse T cell panel, MLE for CD3ε is given by the percentage of CD3ε+CD4+, CD3ε+CD4+FoxP3+ and CD3ε+CD8α+ cells relative to the total number of CD3ε+ cells. Similarly, MLE for CD8α is given by the percentage of CD3ε+CD8α+ cells relative to the total number of CD8α+ cells (see methods for details). The numbers and types of cell phenotypes included in the definition of MLE can vary and depends on the specific questions being asked. Here we have calculated the MLE using the 3 different T-cell phenotypes that we have detected in the multiplex images. MLE values can range from 0 to 100%. For example, an MLE of 100% for CD8α means that the CD3ε biomarker was simultaneously localized in all CD8α+ cells that were detected. An MLE of 90% for CD3ε would indicate that 10% of the CD3ε+ cells labeled were not included among the various T cell phenotypes that were analyzed. In a practical setting, the MLE can be calculated from the positive or run control sample and compared across different runs. We do not recommend calculating MLE from study specimens, especially if changes in biomarker expression levels are anticipated to result from experimental conditions (e.g., drug treatment).

**Figure 5:**
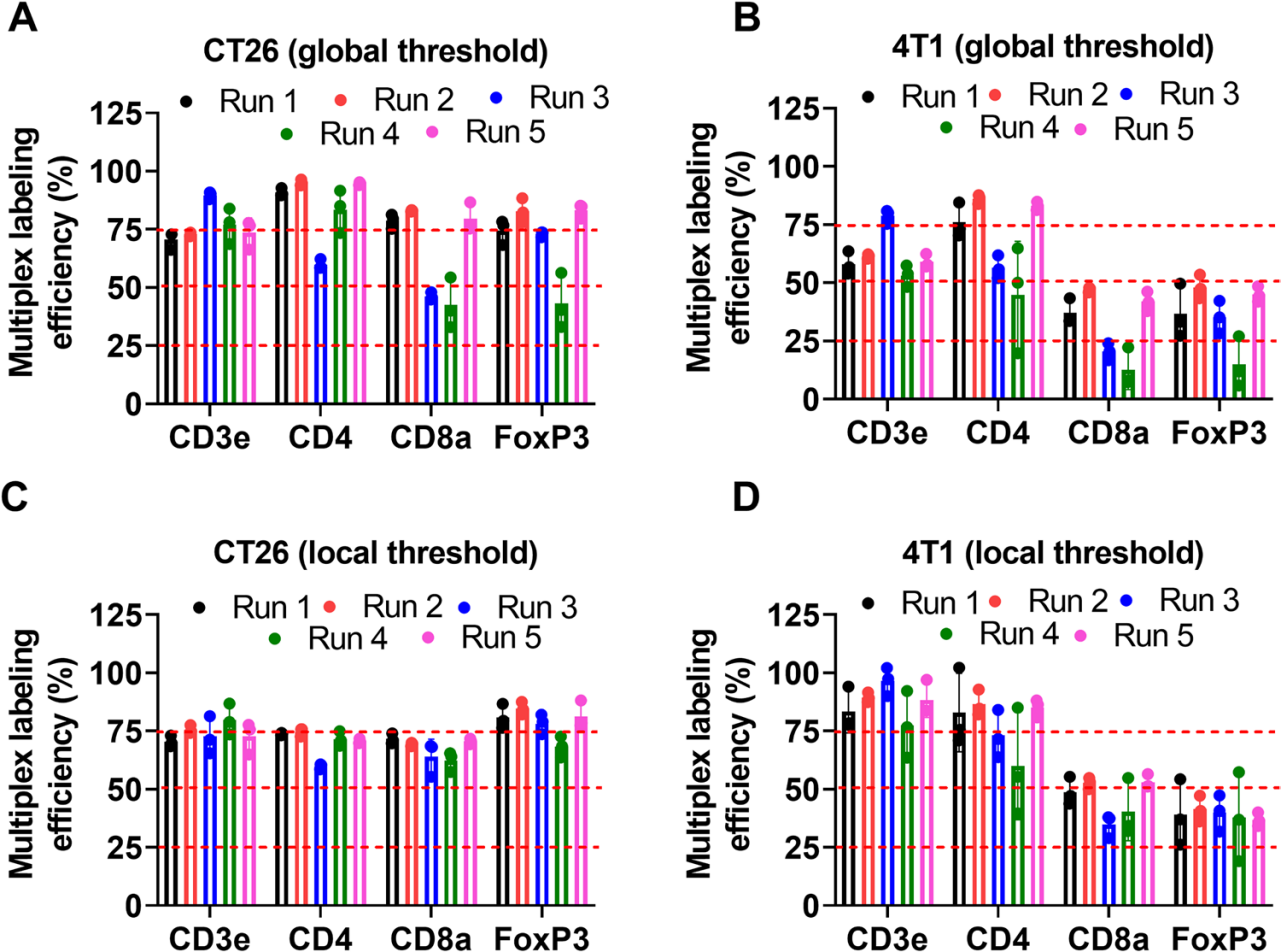
Multiplex labeling efficiency. (**A**) and (**B**) Show the multiplex labeling efficiency calculated for different biomarkers from CT26 and 4T1 tumor images, respectively, where a global thresholding strategy was used to threshold for biomarker positivity. (**C**) and (**D**) Show the multiplex labeling efficiency calculated for different biomarkers from CT26 and 4T1 tumor images, respectively, where local thresholding was used to threshold for biomarker positivity.

Figure 5A,B show MLE calculated for the CT26 and 4T1 tumor models, respectively, where global thresholding was used for each biomarker to define positivity. MLE was relatively constant within a run but showed considerable variability across runs when global thresholding was employed. Figure 5C,D show the MLE for the CT26 and 4T1 models, respectively, where local thresholding was used to define biomarker positivity. In contrast to the results shown in Fig. 5A,B, MLE for a given biomarker was consistent within and across different runs when local thresholding was used. Note that with local thresholding, the MLE for CD3ε and CD4 are relatively consistent at ~ 75% in both CT26 and 4T1 tumor models. However, the MLE for CD8α and FoxP3 is consistently lower (ranging from 30 to 50%) in the 4T1 tumor model relative to CT26 tumor model, where MLE is > 70% (see Supplementary Table 4 for summary table). This difference can be partly attributed to the fact that these biomarkers are expressed by other cell types that were not included in the multiplex panel (e.g., CD8α is also expressed by dendritic cells and NKT cells). As the relative abundance of specific phenotypes of immune cells can vary among tumor models^40,41^, the relative fraction of cells expressing a biomarker of interest alone can also differ among tumor models, which in turn impacts the MLE. Thus, in a practical setting the MLE can be calculated from just one tumor model which is included as a positive control in each run. To flag a particular multiplex run as inaccurate will require a priori information regarding the range of acceptable MLE estimates, which can be deduced from a reproducibility study. Then a routine multiplex IF assay will be flagged if the MLE estimate from that run falls outside the acceptable range. In our current study, the CV of the MLE estimates varies from 5 to 20% depending upon the biomarker. Thus a maximum deviation of ± 10% in the MLE can be used as a cutoff for flagging a multiplex IF run for the mouse panel.

### Precision analysis of cell–cell distance estimates

In many applications, spatial analysis of multiplex data represents a critical endpoint that has significant mechanistic implications. Central to most spatial analysis workflows is the calculation of cell–cell distances which is extensively used to define cellular neighborhoods and to calculate cell proximity to other cell types, structures (e.g., vessels) or functional boundaries (e.g., tumor margins). Because distance estimates are typically calculated from a single histological section of the tissue of interest, a fundamental concern arises with regards to the reproducibility of spatial distance estimates from multiplex data. Here, we made use of precision assay data to quantify the CV of cell-to-cell distance estimates from CT26 and 4T1 tumor specimens, specifically calculating the cell-to-cell distance between regulatory T cells (T_reg_) and cytotoxic T cells (T_cyt_).

**Figure 6:**
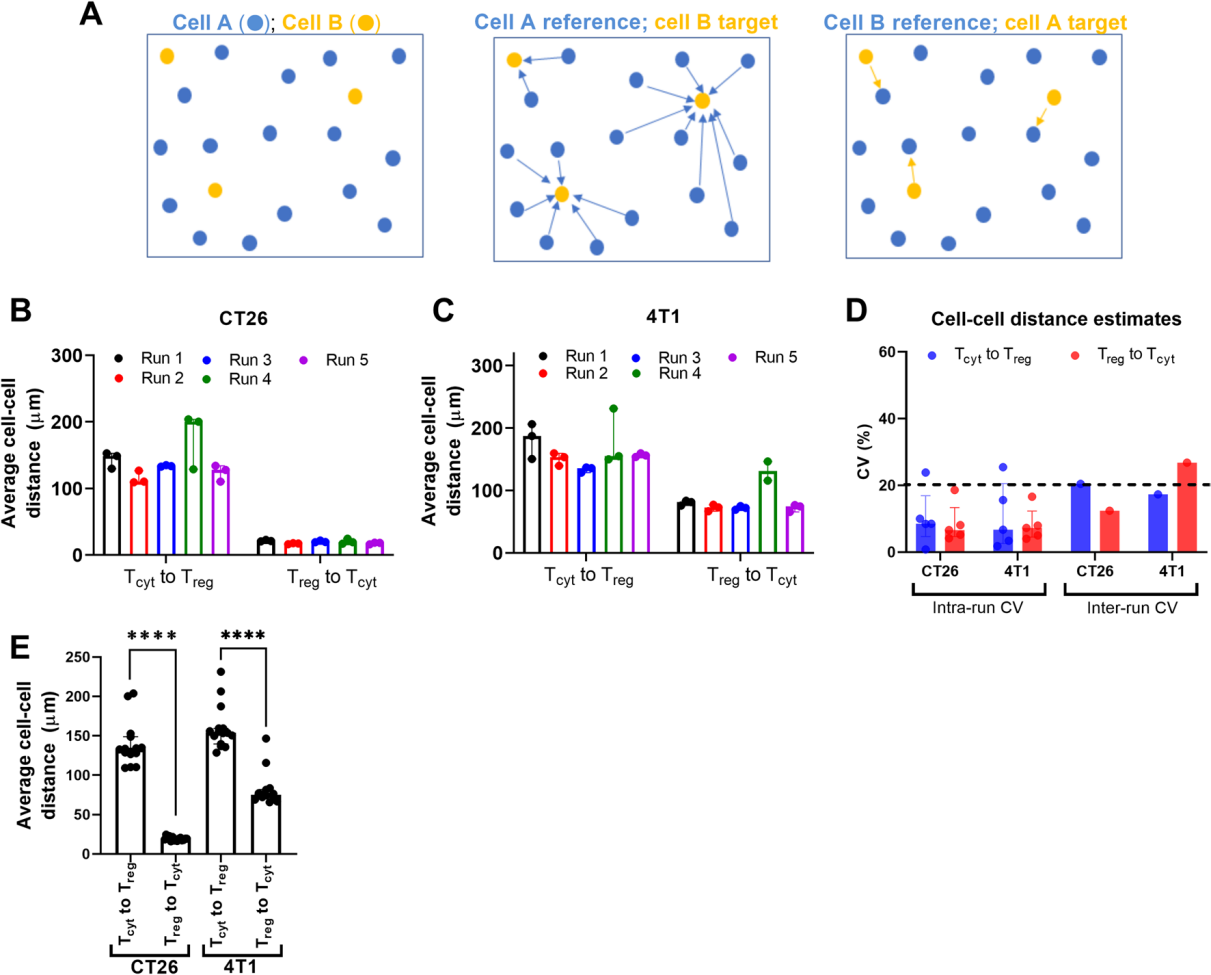
Reproducibility of cell–cell distance estimates in murine tumors. (**A**) Left image shows a hypothetical spatial distribution of two different cell types, i.e., A and B. The middle and right images show two scenarios where one of the cell types is the target and the other cell type is the reference. The arrows indicate the distance between a given reference cell and different target cells. Depending upon the distribution and abundance of the cell types, the choice of reference versus target will affect the numerical value of the cell–cell distance measurement. (**B**) and (**C**) Show the cell–cell distance estimates from CT26 and 4T1 tumor models, respectively, across all the runs. (**D**) Shows the CV of cell–cell distance estimates for CT26 and 4T1 tumor models. (**E**) Shows the average T_cyt_ to T_reg_ and T_reg_ to T_cyt_ distance estimates measured for the CT26 and 4T1 tumor models. ****p < 0.0001.

Figure 6A shows a hypothetical spatial distribution for 2 different cell types A and B. For a given pair of cell types, two different cell–cell distance estimates can be calculated depending upon which phenotype is selected as the reference versus target cell. For instance, cell A can be used as reference to calculate the shortest distance between a given cell A and a cell B, which we refer to as A to B distance. Alternately, we can use cell B as reference and calculate the shortest distance between a given cell B and a cell A, which we refer to as B to A distance. In general, the average of all the A to B distance estimates will be different from the average of B to A distance estimates. For the cell distribution shown in Fig. 5A, the average A to B distance will be greater than the average B to A distance since there are far fewer B type cells relative to A type cells. Thus, on average for a cell B there exists a cell A in proximity due to the relative high density of A type cells (Fig. 6A; right panel). However, the converse is not true, since for a given cell A the nearest cell B is much farther away (Fig. 6A; middle panel). Figure 6B,C show the T_cyt_ to T_reg_ and T_reg_ to T_cyt_ distance estimates for the CT26 and 4T1 tumor models, respectively. The average cell–cell distance estimates were relatively constant within and across different runs for both tumor models (also see Supplementary Table 5 for summary table). This is also reflected in the CV calculations for the cell–cell distance estimates that are shown in Fig. 6D, where the CV of the cell–cell distance estimates was typically less than 20%. In both tumor models the median T_cyt_ to T_reg_ distance was significantly higher than the median T_reg_ to T_cyt_ distance (Fig. 6E). Taken together these results suggest that cell–cell distance estimates obtained from murine tumor sections are highly reproducible.

### Evaluation of human Ultivue 4-plex panels

**Figure 7:**
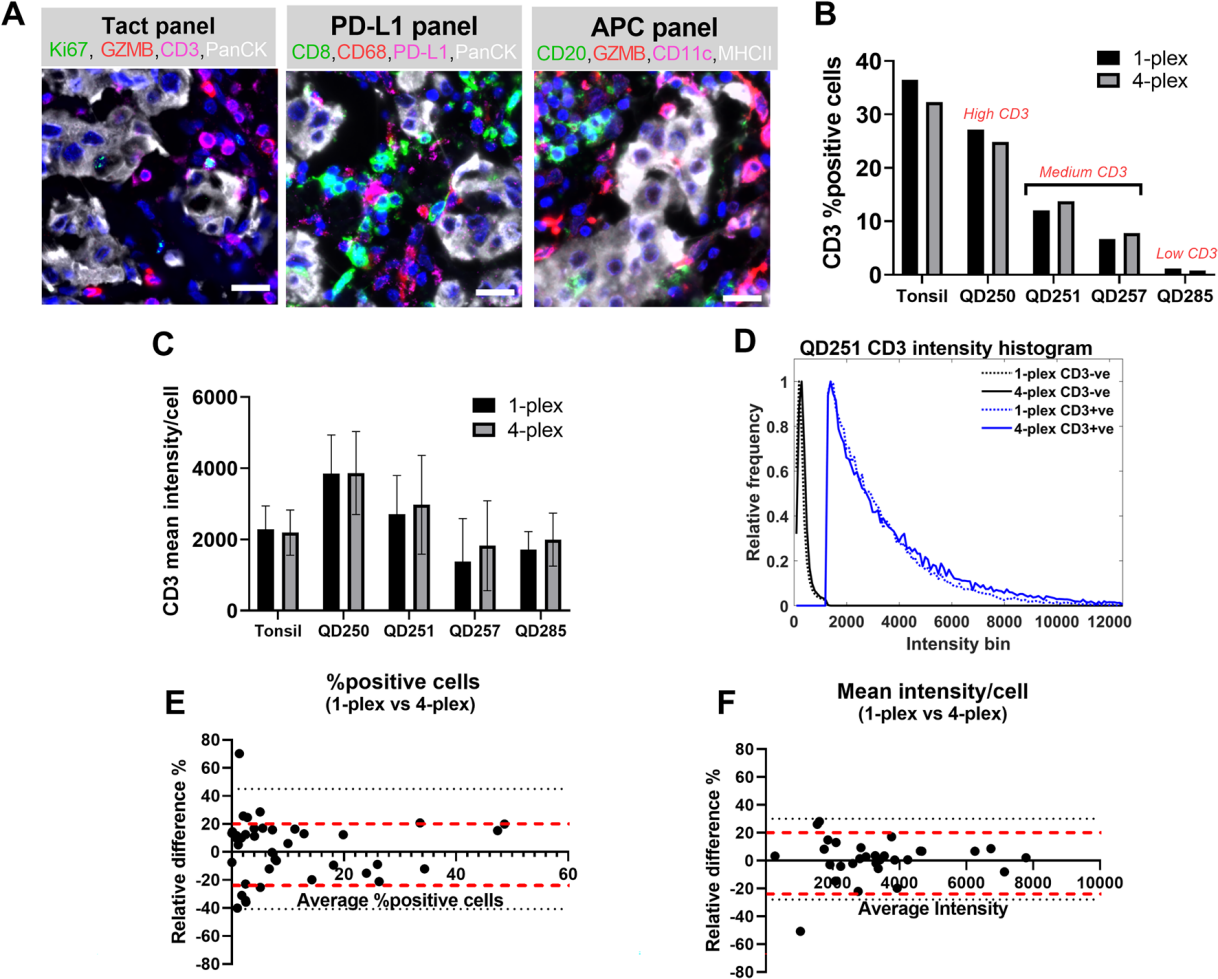
Human 4-plex panels: concordance assay. (**A**) Shows representative images illustrating the immunolabeling pattern of Ultivue Tact, PD-L1 and APC panels in human breast tumor resections. (**B**) Shows the % positive cells for CD3 biomarker obtained from the 1-plex and 4-plex images in 4 different breast tumor resections with varying levels of T cell density. (**C**) Shows the mean CD3 intensity/cell from the 1-plex and 4-plex images in 4 different breast tumor resections with varying levels of T cell density. (**D**) Shows the CD3 intensity histogram for CD3-positive and CD3-negative cells from the 1-plex and 4-plex images for one of the breast tumor resections. (**E**) and (**F**) Show Bland–Altman plots for the relative difference in % positive cells and mean intensity/cell, respectively, for all the unique biomarkers in the 3 human Ultivue panels that were evaluated using 5 different breast tumor specimens.

In addition to analyzing a custom multiplex panel we sought to evaluate the performance of commercially available Ultivue panels. We chose triple negative breast tumor resection specimens as the test substrate and selected three panels (Tact, PD-L1 and APC) directed against human antigens that identify and localize cell populations that are important to the pathogenesis and treatment of neoplastic diseases. These panels detect the following biomarkers (see Fig. 7A): Tact panel: Ki67, CD3, Granzyme B and panCK; PD-L1 panel: CD8, CD68, PD-L1 and panCK; and APC panel: CD11c, CD68/CD163 cocktail, CD20 and MHCII. In the APC panel, the CD68/CD163 cocktail is a combination of anti-CD68 and anti-CD163 antibodies that was found to provide consistent labeling of macrophages. Both antibodies were conjugated to the same DNA barcode and thus would be detected by the same fluorescent probe, which was conjugated to the complementary barcode. The vendor provided the antibody clone information used in their panels upon request. To assess the performance of the antibody clones, we searched for antibody validation reports in the NordiQC consortium database^42^ which provides multi-laboratory testing and evaluation assessment of commonly used antibody clones against human antigens in clinical laboratory practice. Out of 11 antibodies, we found validation reports for 8 antibody clones (CD8, CD68, panCK, CD3, Ki67, CD20, CD68, and CD163) which were all categorized as good for the Leica Bond instrument.

### Concordance assay for the human Ultivue panels

Analogous to the mouse Ultivue panel, we ran concordance and precision assays to assess the accuracy and reproducibility, respectively, of the human multiplex panels. For the concordance assay, we considered 4 different breast tumor resections with differing relative levels of CD3+ T cell density (i.e., containing high (n = 1), medium (n = 2) and low (n = 1)) as assessed through IHC (data not shown).

Figure 7B shows the results of the concordance assay for CD3. The % positive cells between the 1-plex and 4-plex images were consistent across all tumor specimens evaluated including the specimen with low CD3+ T cell infiltration,for which the T-cell abundance is ~ 20-fold lower than the specimen with high CD3+ cell infiltration. Figure 7C shows the mean fluorescence intensity/cell for CD3 which was consistent between the 1-plex and 4-plex images in all the samples (Fig. 7D; also see Supplementary Table 6 for summary table). As breast tumor resections are known to have high levels of autofluorescence we also plotted the relative frequencies of marker-positive and marker-negative cells, shown as histograms of the mean intensity/cell of CD3 in CD3+ and CD3-cells in the 1-plex versus 4-plex images. The intensity histogram for CD3+ cells is a measure of the signal of interest, while the intensity histogram for CD3- cells is a measure of the background. The intensity histograms of CD3+ cells from the 1-plex and 4-plex images show strong overlap, suggesting that the intensity distribution of CD3 is identical in the two images. Similar results were also observed for CD3-cells. It is worth noting that the histograms of CD3+ and CD3- cells are well separated, which suggests good signal-to-background delineation. Similar results were also seen for the other biomarkers that we evaluated in the human 4-plex panels.

Figure 7E shows a Bland Altman plot of the relative difference in % positive cells between 1-plex and 4-plex images in the concordance assay. The relative difference lies predominantly within ± 20% for all the biomarkers examined. The high numerical values for relative difference % are typically for biomarkers that have very low abundance in the tumor sections, and therefore exhibit higher variability in cell counts between adjacent serial sections. Figure 7F shows a Bland Altman plot of the relative difference in the mean intensity/cell between 1-plex and 4-plex images. Analogous to Fig. 7E, the relative difference is typically within ± 20% for all the biomarkers. Taken together, these results suggest that there is robust concordance between the 1-plex and 4-plex images for each of the biomarkers evaluated for the human Ultivue panels.

### Precision assay for human Ultivue panels

**Figure 8:**
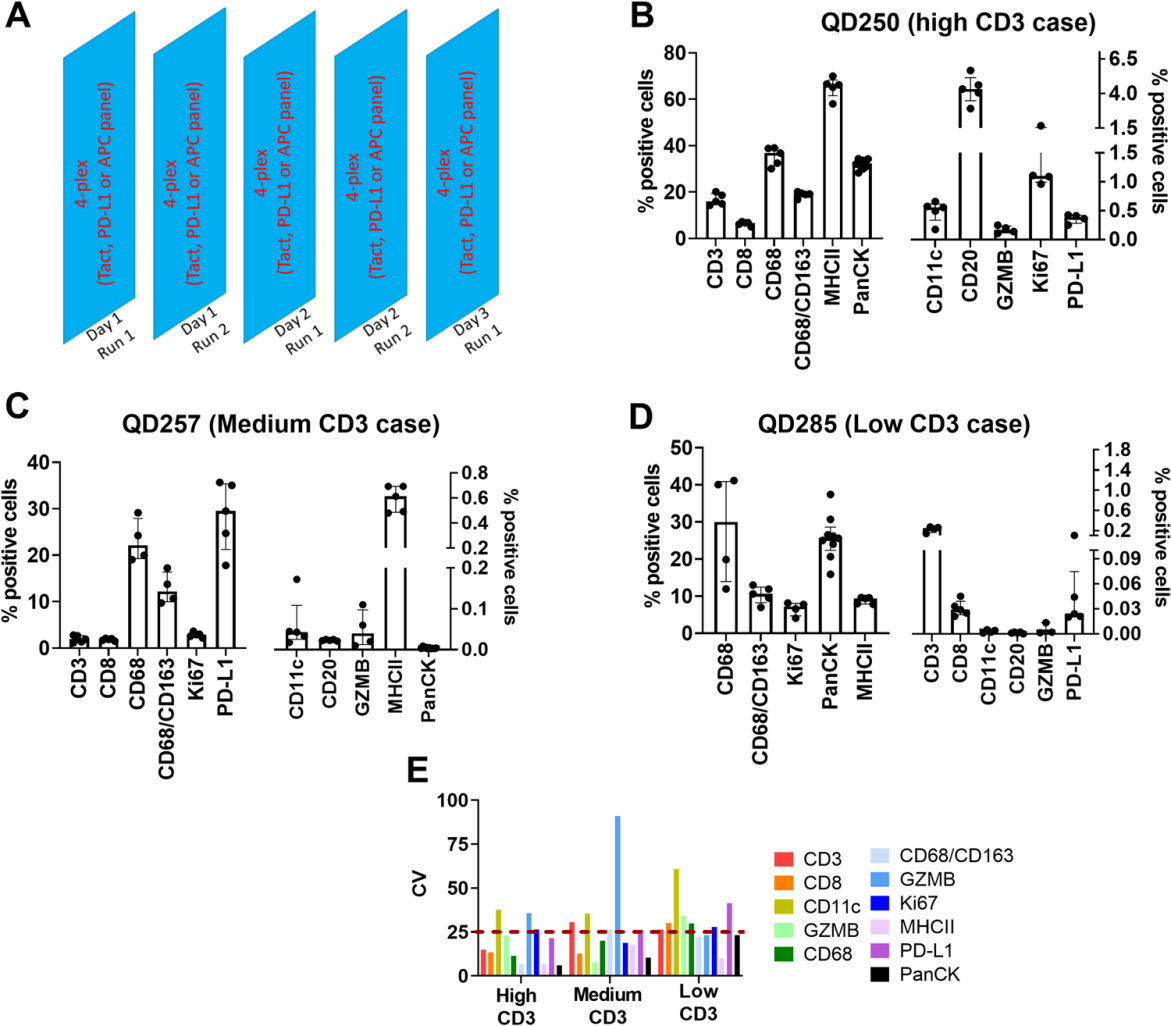
Human 4-plex panels: reproducibility assay. (**A**) Shows the study design of the human reproducibility assay for the human 4-plex panels. For each multiplex panel, 3 breast tumor resections with high, medium, and low relative T cell densities were selected and the panel was run independently at 5 different times. (**B**), (**C**) and (**D**) Show the % positive cells for each biomarker from the breast tumor specimens with high, medium, and low relative CD3 densities, respectively. In each panel, the data is plotted against two separate y-axis frames with different scales. (**E**) Shows the inter-run CV of % positive cells for the single-marker cell phenotypes from all the specimens used in the reproducibility study.

We next evaluated the reproducibility of the human Ultivue panels. Figure 8A shows the schematic of the precision assay, in which five separate runs were performed for each multiplex panel. In each run three breast tumor resections were assayed, including one each containing high, medium and low relative numbers of infiltrating CD3+ T cells. Analogous to the mouse panel, we calculated % positive cells for individual biomarkers to assess the reproducibility of the human Ultivue panels. In addition, the serial sections were shuffled such that sequential sections were not used in consecutive runs and local thresholding was used to analyze the images from each run. Figure 8B–D show the % positive cells for the single-marker cell phenotypes for the specimens containing high-, medium- and low-density CD3+ T cells, respectively. The % positive cells was generally consistent across all runs for a given single-marker cell phenotype (also see Supplementary Table 7 for summary table). Figure 8E depicts the CV of % positive cells for the single-marker cell phenotypes for all biomarkers tested and shows that the CV is typically ~ 25% for most of the biomarkers, although there are a few exceptions. For example, the CV of % positive cells for Granzyme B (GZMB) is ~ 80% for the medium CD3 specimen. This can be partly attributed to the fact that GZMB expression was relatively low in these tumor specimens which introduces relatively high variability in GZMB+ cell counts between serial sections, resulting in a high CV. The CV of % positive cells for CD11c was also consistently higher than 25% in all specimens, likely attributable to the same phenomenon.

**Figure 9:**
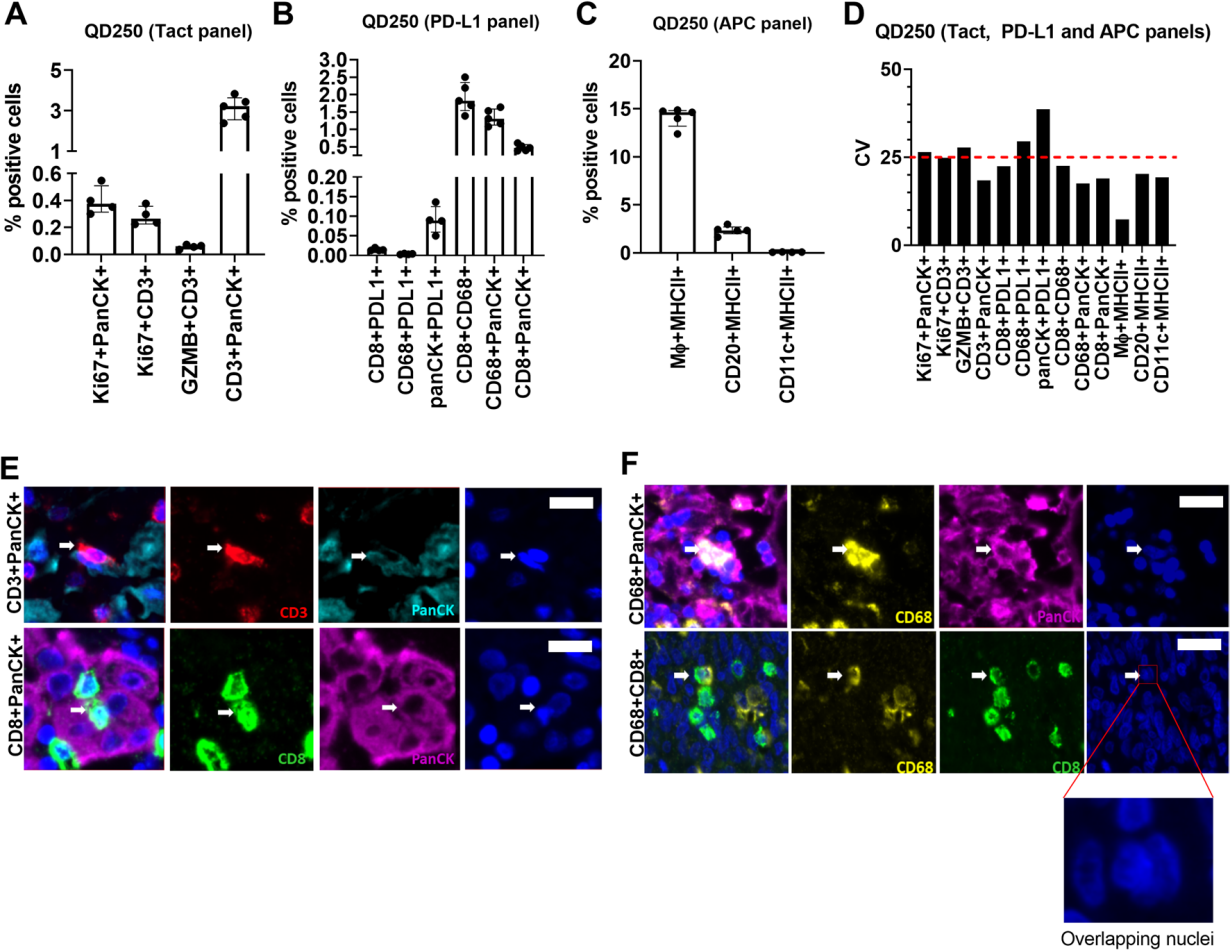
Reproducibility of multi-marker cell phenotypes in human 4-plex panels. (**A**), (**B**) and (**C**) Show the % positive cells for multi-marker cell phenotypes obtained from the breast tumor resection with high relative CD3 density (QD250) for the Tact, PD-L1 and APC panels, respectively. (**D**) Shows the inter-run CV of % positive cells for the multi-marker cell phenotypes across all the multiplex panels for the breast tumor resection with high relative CD3 density. (**E**) Shows representative images of CD3+and CD8+ cells that are in close contact with PanCK+ cells, which are detected as single cells by the image analysis algorithm. (**F**) Shows representative images of PanCK+ cells and CD8+ cells in close contact with CD68+ cells. Inset shows an example of how cell–cell couples are detected as single cells due to considerable nuclear overlap, resulting in the detection of cells with atypical combinations of biomarkers. Scale bar equals 25 microns in (**E**) and (**F**).

Figure 9A–C show the % positive cells for multi-marker cell phenotypes obtained from the tumor specimen with high numbers of CD3+ T cells for the Tact, PD-L1 and APC panels, respectively. Consistent with the single-marker phenotypes, the % positive cells for the multi-marker phenotypes were generally consistent across different runs (also see Supplementary Table 8 for summary table). This is also reflected in the CV of % positive cells which is less than 30% for 12 of the 13 multi-marker cell phenotypes evaluated (Fig. 9D).

Interestingly, in each of the panels we observed unusual combinations of biomarkers that are generally not expressed on the same cell populations. For example, in the Tact panel we observed ~ 3% of cells to be CD3+PanCK+. Similarly, in the PD-L1 panel, we observed CD68+panCK+ cells, CD8+CD68+ cells and CD8+PanCK+ cells. On closer examination of the multiplex images, it was discovered that cells displaying these unusual phenotypes were, in fact, distinct cells that were in contact with one another (Fig. 9E,F). Specifically, the CD3+PanCK+ and CD8+PanCK+ phenotypes identify T cells that are in close proximity with tumor cells, while the CD68+PanCK+ phenotype localizes CD68+ cells that are in close contact with tumor cells and the CD68+CD8+  phenotype recognizes T cells interacting with macrophages. Because of close spatial proximity, these cell–cell couples were detected as single objects by the image analysis algorithm and recorded as unusual phenotypes. The relatively low CV of these unusual phenotypes suggests that these cell–cell interactions are seen in multiple runs and are not an isolated observation.

## Discussion

The advent of tissue multiplexing technology has created a new field of spatial biology which holds the promise to deliver novel insights into the cellular microenvironments within tissue sections. While the promises of this technology are great, so are its challenges. Foremost, multiplexing technology needs to be adequately validated and qualified as fit for purpose for the intended application. Despite the explosive growth of multiplexing techniques introduced in the past decade, there is a severe paucity of data concerning the accuracy and precision of these technologies. Here, we have presented a rigorous, quantitative strategy to validate tissue multiplexing panels by performing whole slide image analysis. Our study was designed to address both accuracy and precision of multiplexing assays, two key concerns regarding the general application of this technology. To our best knowledge, this work represents the first report of a multi-sample validation strategy, which we applied to validate Ultivue InSituPlex multiplex technology, using three off-the-shelf human 4-plex panels and one custom mouse 4-plex panel. Tumor tissue specimens with varying levels of T cell density were used as an appropriate substrate for these assay validation experiments.

Our observations of the tight concordance in the mean intensity/cell and in % positive cells between the 1-plex and 4-plex images for all biomarkers evaluated suggests that Ultivue Insituplex is a robust technology that does not affect antigen–antibody interactions. It also underscores the modular nature of this methodology, in which antibodies directed against different biomarkers can be mixed and matched. Not surprisingly, the FlexVUE™ panel, which is currently offered by Ultivue, facilitates on-demand creation of custom multiplex panels from a large selection of validated antibodies. Our results from the concordance study for the murine 4-plex panel are consistent with our previous report that investigated intra-tumor variability in immune cell abundance in murine tumor specimens^34^, in which we reported a CV of ≤ 20% in the abundance of immune cell populations quantified from IHC images of adjacent serial sections of murine tumors. Our observation that the relative difference in % positive cells between 1-plex and 4-plex images in this study was typically less than 20% suggests that this variability can largely be attributed to tumor-intrinsic variance rather than variability in the multiplex panel. Similar observations in the relative difference in % positive cells detected using the human panels suggests that this variability is likely to be sample-intrinsic for human biomarkers as well.

Our design of the precision study for the mouse multiplex panel facilitated evaluation of both intra-run and inter-run variability. The serial sections used for the precision assay were shuffled such that no two adjacent serial sections were included in the same run. In this way we minimized systematic drifts in the precision study that could arise when adjacent serial sections are sequentially used in subsequent runs. Additionally, all reagents used for the precision study were from the same lot, which ensured that the quality of labeling achieved with the antibodies and the readout probes were similar across all runs. Moreover, the same slide scanner was used to scan all the slides from the precision study. Despite these steps, our observations of low intra-run variability (CV ≤ 25%) and high inter-run variability (CV >> 25%) raises several questions regarding the run-to-run reproducibility of multiplex panels.

Significantly, our analysis revealed that there was considerable variation in the signal intensity of the biomarkers across different runs in the precision study. This resulted in a high inter-run CV for the mouse panel when a global thresholding strategy was used, wherein the same intensity threshold for biomarker positivity was applied across all images from all runs in the study. Interestingly, a local thresholding strategy for biomarker positivity mitigated the high inter-run CV without affecting intra-run CV.

For the human panels, the precision study design did not include replicates within a given run due to cost considerations. In this instance, we evaluated the reproducibility of three different multiplex panels by including 3 specimens with varying levels of T cell infiltration in each run for each panel. This resulted in a threefold increase in the number of slides in the precision study relative to the mouse panel. Based on our prior experience with the mouse panel, we analyzed the precision data of the human panels using a local thresholding strategy. Our observations that the inter-run CV for most biomarkers was relatively low suggests that the human multiplex panels exhibit robust reproducibility. It is worth noting that run-to-run variability in biomarker intensity is not limited to Ultivue methodology but is a broader problem for multiplexing technologies, as evidenced by the application of normalization strategies to mitigate batch-to-batch variation, as described^22,43–45^ in the analysis of other multiplex datasets.

We note that our choice of CV cutoff value of 25% is consistent with prior reports^32–35^ that used a similar CV cutoff value to assess the reproducibility of IHC assays. We recognize that the choice of the cutoff value is arbitrary and that a lower cutoff value provides a more stringent performance measure. Indeed, many of our intra-run and inter-run CVs were in the 10–15% range. In our case, the rationale for selecting 25% was to accommodate the higher variability that intrinsically arises when detecting very low abundance biomarkers and cell types. Although CV is a widely used metric to assess the reproducibility of IHC assays, it may not be suitable in some cases when the number of replicates varies between experiments. In such instances, the use of a robust metric of dispersion (for example, standard error%) is necessary^46^. In our case, the number of replicates was the same since the comparison of CV was made between different biomarkers from the same reproducibility study.

In many practical situations the inclusion of technical replicates to compute intra-run CV is not feasible. This raises the question of how run-to-run variability can be monitored from multiplex data. The MLE metric that we have introduced here provides a simple, yet useful endpoint to assess run-to-run variability; for example, when global intensity thresholding is used for gating biomarker positivity. We recommend the calculation of MLE from a positive control sample. An advantage of MLE is that it tracks the intensity of multiple biomarkers which define cell positivity; thus, this metric provides a comprehensive assessment of multiple biomarkers. A limitation of MLE is that its calculation is typically informative of biomarkers that are restricted to labeling one or a few cell types. For instance, if the biomarkers in the multiplex panel label different cell phenotypes, then the calculation of MLE may not be informative. This was one of the reasons why we did not attempt to calculate the MLE for the human Ultivue panels, since the biomarkers in the different panels label distinct cell types.

The spatial disposition and relationships among different cell phenotypes represent a critical endpoint of multiplex imaging data. Despite significant interest in spatial analysis, there is a paucity of data concerning the reproducibility of distance estimates from multiplex images. Tumor specimens are inherently three-dimensional objects; thus, the distance measurements from multiplex data represent two-dimensional estimates of true cell–cell distances. Nevertheless, our observations that the CV of cell–cell distance estimates in multiple murine tumor models is relatively low suggests that these estimates are robust across serial sections. Moreover, our results also imply that a single tumor section is adequate to estimate two dimensional cell–cell distances. In most practical situations, cell–cell distance estimates are used to assess mechanistic insights, for example, to determine whether the cells move closer or farther apart from one another due to test-article treatment. It is well known that cell–cell distance estimates depend on the relative abundance of cell types, and the shape and size of the tissue section from which the estimates are computed^47,48^. Thus, it is critical to carry out statistical permutation tests to rule out the effects of these confounding factors and determine whether the observed distance estimates are biologically relevant^47,48^.

In the human panels, our workflow detected unusual cell phenotypes, that is, cells positive for distinct cell-lineage biomarker combinations such as CD3+PanCK+ cells and CD8+PanCK+ cells. Visual inspection of the multiplex images revealed that these phenotypes arise due to the proximity of distinct cell phenotypes, which highlights a limitation of the nuclear/cell segmentation algorithm that was used to analyze the images. Specifically, our nuclear segmentation algorithm relied on traditional approaches which could not recognize overlapping nuclei. We anticipate that the use of deep learning-based segmentation algorithms could mitigate this issue. This also underscores the importance of visual review of the images, for example, when automated clustering algorithms are used to infer cell phenotypes.

Detection of unusual cell phenotypes also arise due to lateral/membrane spillover which occur when there is cell–cell contact between distinct cell types (e.g., CD3ε+ cell touching a CD20+ cell), for example, in crowded cellular environments such as lymphoid clusters. Specifically, the close juxtaposition of the cell membranes combined with imprecise nuclear/cell segmentation typically gives rise to this artefact. Recently, several groups have proposed spillover correlation strategies to mitigate this effect. In Ref^9^, the authors proposed a linear correction algorithm that is analogous to compensation correction in flow cytometry. In Ref^49^, the authors developed a novel unsupervised compensation algorithm that is compatible with fluorescence and mass cytometry based multiplexing techniques. It is worth noting that none of the above approaches can handle the scenario when the cells overlap with each other.

In conclusion, we present a comprehensive strategy to validate multiplex panels and have applied it to evaluate the accuracy and precision of Ultivue InsituPlex technology. We evaluated 4 different Ultivue panels using multiple tumor specimens with varying levels of T cell density. Our results reveal that Ultivue IF panels show robust performance in concordance and precision. We also report the reproducibility of cell–cell distance estimates which exhibit relatively low intra-run and inter-run CV in specimens from two different murine tumor models. Finally, we introduce a new metric, multiplex labeling efficiency, to benchmark the performance of a multiplex panel and to track batch-to-batch variability. Our results and analyses provide a template to validate and benchmark multiplex panels and offer guidelines for analyzing multiplex imaging data.

### Supplementary Information

Supplementary Table S1.

Supplementary Table S2.

Supplementary Table S3.

Supplementary Table S4.

Supplementary Table S5.

Supplementary Table S6.

Supplementary Table S7.

Supplementary Table S8.

Supplementary Legends.

## Data Availability

The datasets used and/or analyzed in this study are available on reasonable request from the corresponding author.
