# Supplementary material for: Quantitative performance assessment of Ultivue multiplex panels in formalin-fixed, paraffin-embedded human and murine tumor specimens
Source: Sci Rep. 2024 Apr 11;14:8496. doi: 10.1038/s41598-024-58372-5 (PMC11009312; doi:10.1038/s41598-024-58372-5)
Supplement: Supplementary file 9 — Supplementary Legends. [file 41598_2024_58372_MOESM9_ESM.docx]

**Supplementary table 1:** The Excel file contains summary tables showing the mean, standard deviation and number of replicates of data shown in the individual plots in figures 2F-2I. No summary table is provided for plots that have no data replicates.

**Supplementary table 2:** The Excel file contains summary tables showing the mean, standard deviation and number of replicates of data shown in the individual plots in figures 3C-3K. No summary table is provided for plots that have no data replicates.

**Supplementary table 3:** The Excel file contains summary tables showing the mean, standard deviation and number of replicates of data shown in the individual plots in figure 4. No summary table is provided for plots that have no data replicates.

**Supplementary table 4:** The Excel file contains summary tables showing the mean, standard deviation and number of replicates of data shown in the individual plots in figure 5.

**Supplementary table 5:** The Excel file contains summary tables showing the mean, standard deviation and number of replicates of data shown in the individual plots in figures 6B-6E.

**Supplementary table 6:** The Excel file contains the summary table showing the mean, standard deviation and number of replicates of data shown in the individual plot in figure 7C.

**Supplementary table 7:** The Excel file contains summary tables showing the mean, standard deviation and number of replicates of data shown in the individual plots in figures 8B-8D. No summary table is provided for plots that have no data replicates.

**Supplementary table 8:** The Excel file contains summary tables showing the mean, standard deviation and number of replicates of data shown in the individual plots in figures 9A-9C. No summary table is provided for plots that have no data replicates.
